# Accelerated atrial pacing reduces left-heart filling pressure: a combined clinical-computational study

**DOI:** 10.1093/eurheartj/ehae718

**Published:** 2024-11-26

**Authors:** Tim van Loon, Jesse Rijks, Johan van Koll, Joey Wolffs, Richard Cornelussen, Nick van Osta, Justin Luermans, Frits Prinzen, Dominik Linz, Vanessa van Empel, Tammo Delhaas, Kevin Vernooy, Joost Lumens

**Affiliations:** Department of Biomedical Engineering, Cardiovascular Research Institute Maastricht (CARIM), Maastricht University, Universiteitssingel 40, 6229 ER Maastricht, The Netherlands; Department of Cardiology, Cardiovascular Research institute Maastricht (CARIM), Maastricht University Medical Center, Maastricht, The Netherlands; Department of Cardiology, Cardiovascular Research institute Maastricht (CARIM), Maastricht University Medical Center, Maastricht, The Netherlands; Department of Biomedical Engineering, Cardiovascular Research Institute Maastricht (CARIM), Maastricht University, Universiteitssingel 40, 6229 ER Maastricht, The Netherlands; Department of Physiology, Cardiovascular Research institute Maastricht (CARIM), Maastricht University, Maastricht, The Netherlands; Medtronic Bakken Research Center, Maastricht, The Netherlands; Department of Biomedical Engineering, Cardiovascular Research Institute Maastricht (CARIM), Maastricht University, Universiteitssingel 40, 6229 ER Maastricht, The Netherlands; Department of Cardiology, Cardiovascular Research institute Maastricht (CARIM), Maastricht University Medical Center, Maastricht, The Netherlands; Department of Physiology, Cardiovascular Research institute Maastricht (CARIM), Maastricht University, Maastricht, The Netherlands; Department of Cardiology, Cardiovascular Research institute Maastricht (CARIM), Maastricht University Medical Center, Maastricht, The Netherlands; Department of Cardiology, Cardiovascular Research institute Maastricht (CARIM), Maastricht University Medical Center, Maastricht, The Netherlands; Department of Biomedical Engineering, Cardiovascular Research Institute Maastricht (CARIM), Maastricht University, Universiteitssingel 40, 6229 ER Maastricht, The Netherlands; Department of Cardiology, Cardiovascular Research institute Maastricht (CARIM), Maastricht University Medical Center, Maastricht, The Netherlands; Department of Biomedical Engineering, Cardiovascular Research Institute Maastricht (CARIM), Maastricht University, Universiteitssingel 40, 6229 ER Maastricht, The Netherlands

**Keywords:** Atrial fibrillation, Heart failure with preserved ejection fraction, Heart rate, Filling pressure, Computer modelling, AV sequential pacing

## Abstract

**Background and Aims:**

Accelerated atrial pacing offers potential benefits for patients with heart failure with preserved ejection fraction (HFpEF) and atrial fibrillation (AF), compared with standard lower-rate pacing. The study investigates the relationship between atrial pacing rate and left-heart filling pressure.

**Methods:**

Seventy-five consecutive patients undergoing catheter ablation for AF underwent assessment of mean left atrial pressure (mLAP) and atrioventricular (AV) conduction delay (PR interval) in sinus rhythm and accelerated atrial pacing with 10 bpm increments up to Wenckebach block. Computer simulations (CircAdapt) of a virtual HFpEF cohort complemented clinical observations and hypothesized the modulating effects of AV coupling and atrial (dys)function.

**Results:**

In the study cohort, 49(65%) patients had a high HFpEF likelihood (H_2_FPEF ≥ 5.0), and 28(37%) an elevated mLAP ≥ 15 mmHg at sinus rhythm. Optimal pacing rates of 100 [70–110]bpm (median [IQR]) significantly reduced mLAP from 12.8 [10.0–17.4]mmHg in sinus rhythm (55 [52–61]bpm) to 10.4 [7.8–14.8]mmHg (*P* < .001). Conversely, higher pacing rates (130 [110–140]bpm) significantly increased mLAP to 14.7 [11.0–17.8]mmHg (*P* < .05). PR interval and, hence, AV conduction delay prolonged incrementally with increasing pacing rates. Simulations corroborated these clinical findings, showing mLAP reduction at a moderately increased pacing rate and a subsequent increase at higher rates. Moreover, simulations suggested that mLAP reduction is optimized when AV conduction delay shortens with increasing rate.

**Conclusions:**

Accelerated pacing acutely reduces left-heart filling pressure in patients undergoing AF catheter ablation and computer simulations with HFpEF features, suggesting it as a potential therapeutic strategy to alleviate congestion symptoms. Virtual HFpEF patient cohorts hypothesize that AV sequential pacing may further optimize this therapy's beneficial effects.

## Structured Graphical Abstract

**Structured Graphical Abstract:**
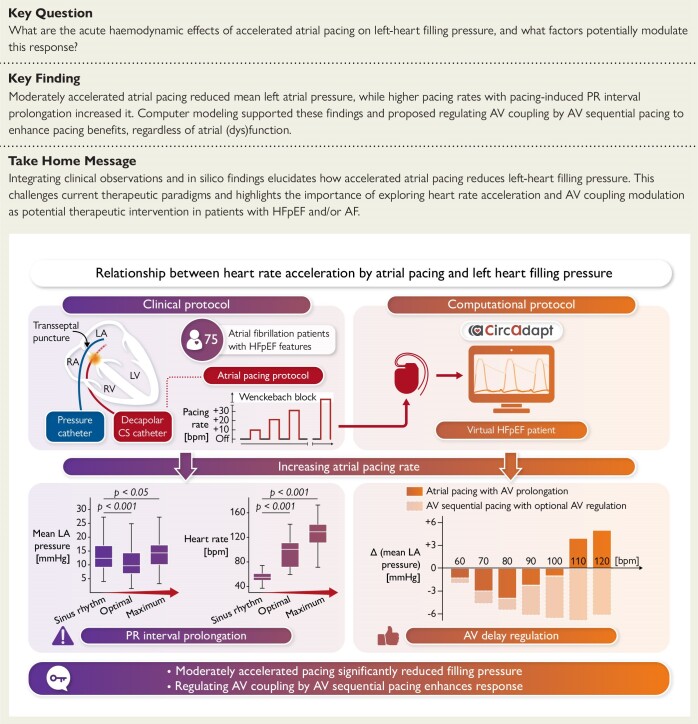
Accelerated pacing reduces left-heart filling pressure, overview of the combined clinical-computational study.

**See the editorial comment for this article ‘Accelerated pacing as a treatment for heart failure with preserved ejection fraction and atrial fibrillation?', by M. Meyer, https://doi.org/10.1093/eurheartj/ehae766.**

**Translational perspective**

Congestion-related symptoms, like dyspnoea and exercise intolerance, often arise from elevated left-heart filling pressure in heart failure with preserved ejection fraction (HFpEF) patients. Despite their high prevalence and morbidity, therapeutic options remain limited. This study demonstrates accelerated pacing as a promising therapeutic intervention to alleviate left-heart filling pressure in atrial fibrillation (AF) patients. Virtual simulations of HFpEF patients support this, suggesting further optimization through atrioventricular (AV) sequential pacing. This challenges current therapeutic paradigms, underscoring the need to explore heart rate acceleration and AV coupling modulation as therapeutic intervention in HFpEF and/or AF.

## Introduction

Despite the high prevalence and severe morbidity, treatment options for heart failure with preserved ejection fraction (HFpEF) remain limited, underscoring the need for novel therapeutic interventions.^1–3^ In this search, the recent myPACE randomized clinical trial has shown that continuous atrial pacing at a moderately increased pacing rate improves quality of life, NT-proBNP levels, physical activity and reduce relative risk of atrial fibrillation (AF) in HFpEF patients with a preexisting pacemaker system, compared with the standard lower-rate setting of 60 bpm.^4^ Moreover, this study provided important evidence for heart rate acceleration as a therapeutic strategy in patients with HFpEF. However, the underlying mechanisms underlying the relationship between heart rate elevation and left-heart filling pressure changes remain incompletely understood. In addition, due to the decremental atrioventricular (AV) nodal conduction properties, increasing atrial pacing rate is associated with PR interval prolongation.^5^ Moreover, a pathological prolonged PR interval has been associated with impaired ventricular filling and higher filling pressures.^6^ Therefore, PR interval regulation through sequential AV pacing may be important in obtaining beneficial therapy response.

In this study, we aimed to elucidate the relationship between accelerated atrial pacing rates and left-heart filling pressure. To this purpose we assessed the invasive haemodynamic effects of accelerated atrial pacing in a clinical study with patients undergoing catheter ablation for AF. Secondly, we used complimentary computational modelling of the human cardiovascular system to hypothesize on the mechanistic and haemodynamic effects of accelerated pacing in a virtual HFpEF cohort with and without sequential AV pacing under controlled *in silico* conditions.

## Methods

### Study population

The study population constituted a consecutive cohort of patients with paroxysmal AF who were scheduled for pulmonary vein isolation (catheter ablation) enrolled in the ISOLATION registry (NCT04342312) at the Maastricht University Medical Center+ (MUMC+).^7^ All invasive measurements required for this study were collected prior to catheter ablation and only when the patients were in sinus rhythm. In the event of AF rhythm during the procedure, the patient was excluded from the study. Clinical characteristics were prospectively assessed in all patients. The execution of the study complied to the principles outlined in the Declaration of Helsinki on research in human subjects and with the procedures of the local Medical Ethics Committee. No additional structural or functional HFpEF inclusion criteria for the accelerated pacing protocol were defined.

### Clinical protocol

**Figure 1:**
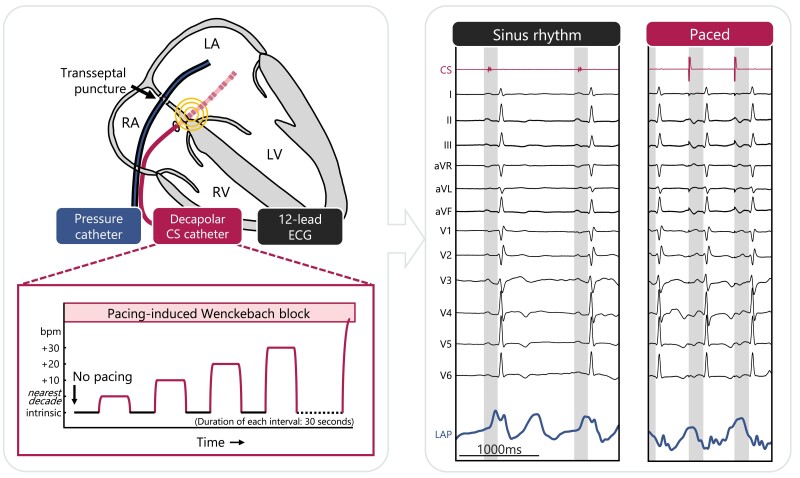
Schematic representation of the clinical protocol. Pressure measurements were obtained using a fluid-filled catheter in the left atrium accessed through septal puncture. A decapolar coronary sinus catheter paced near the interatrial septum. Pacing rate was gradually increased from the nearest decade above the patients-specific intrinsic heart rate to pacemaker-induced Wenckebach block, in increment of 10 beats per minute, with 30-s intervals of pacemaker on and off. In the offline data analysis, the acute changes in mean left atrial pressure and PR interval (shaded areas) induced by accelerated pacing were investigated with respect to sinus rhythm (100 bpm vs. 52 bpm, respectively). LV, left ventricle; RA, right atrium; RV, right ventricle

*Figure 1* provides a schematic representation of the clinical protocol. In summary, patients underwent catheter ablation under either general anaesthesia or sedation. All instrumentations used for this study were routine procedures for catheter ablation, including invasive LA pressure monitoring. First, a coronary sinus (CS) multipolar EP catheter was positioned through femoral vein access. Then, a transseptal atrial puncture was performed under transoesophageal echocardiography guidance using a fluid-filled SL0 sheath connected to a pressure sensor (Abbott Medical, IL, USA). After calibrating the pressure catheter, the accelerated atrial pacing protocol was performed from the proximal electrodes on the decapolar CS catheter, near the interatrial septum (*Figure 1*, top left).

The accelerated atrial pacing algorithm consisted of an alternating protocol of 30 s each, switching between no pacing (intrinsic heart rate in sinus rhythm) and accelerated atrial pacing rates with 10 bpm increments (*Figure 1*, bottom left). The alternating and incremental pacing cycle continued until pacing-induced Wenckebach block. The 30-s intervals were specifically chosen to allow for haemodynamic stabilization after changing pacemaker setting.^8^ The LA pressure, standard 12-lead ECG, and decapolar CS catheter recordings were digitized at a sampling frequency of 1000 Hz, automatically synchronized, and stored using the LSPRO-BARD system (Boston Scientific, MA, USA).

### Data analysis

To reduce transient effects of pacemaker setting changes and allow for haemodynamic stabilization by the autonomic nerves system, our analysis of mLAP was limited to the final 10 s of each 30-s interval in the alternating pacing protocol. The ECG recordings were filtered to reduce noise using a bandpass filter (.5–100 Hz) and an IIR filter designed to reject 50 Hz powerline noise.^9^ Additionally, a moving-median filter (800 samples) was applied to correct for possible baseline drift.

Since the purpose of the clinical study was to assess the acute haemodynamic and electrical effects of accelerated atrial pacing compared with sinus rhythm, the synchronized LA pressure and ECG recordings were divided into two categories: intrinsic (non-paced in sinus rhythm) and paced beats (*Figure 1*, right panel). The paced beats were further grouped based on pacing rate. The change in left-heart filling pressures for each pacing rate was taken as the difference of the average mean left atrial pressure (mLAP) during pacing compared with the average mLAP at sinus rhythm prior to and after each pacing interval. This approach reduces potential carry-over effects from previous pacing sequences and accounts for changes in baseline haemodynamics throughout the procedure.

Electrical effects of accelerated pacing were assessed by calculating and comparing PR intervals between intrinsic sinus rhythm and paced beats, with R-peaks detected using the Pan-Tompkins algorithm.^10^ PR interval, defined as the interval between QRS complex onset and P wave onset for intrinsic beats or atrial pacing spike onset for paced beats, was estimated using MATLAB R2019a (MathWorks, MA, USA) and manually verified.

### Computer simulations

The CircAdapt biophysical model of the heart and circulation^11,12^ was used to study the haemodynamic effects of accelerated pacing in a virtual patient cohort with more pronounced HFpEF features as compared with our patient cohort. The model consists of modules representing cardiac walls, cardiac valves, large blood vessels, systemic and pulmonary peripheral vasculature, the pericardium, and local passive and active tissue cardiac myofiber mechanics. This well-controlled and well-validated in silico environment allows precise model parameter variation and has been shown to accurately replicate (patho-)physiological conditions, including LV diastolic dysfunction^13^ and atrial myopathy,^14^ and has been extensively validated to realistically simulate cardiac mechanics and haemodynamics during various pacing strategies.^15,16^ The model's biophysical foundation enables exploration of cause-effect relationships between tissue abnormalities and cardiac pump function. By changing the model parameter representing heart rate and AV delay, a virtual pacing protocol is simulated to predict the change in mLAP which is a direct result of the underlying biophysical laws and physiological principles incorporated in the CircAdapt model. This approach allowed us to hypothesize on the key mechanisms of action that are not directly observable or testable in clinical setting.

First, mechanics and haemodynamics of virtual patient cohort with structural and functional HFpEF features was simulated, as previously published.^13^ In brief, by impairing LV active relaxation function and increasing LV passive stiffness and wall mass, the cohort captures key functional and structural HFpEF features, including preserved LV ejection fraction, LV concentric hypertrophy, and pseudonormal diastolic dysfunction (E/A ratio: 1.1), dilated LA (volume: 108 mL), and elevated filling pressures in sinus rhythm (mLAP: 15.0 mmHg). Simulated sinus rhythm is set to 54 bpm. Using the homeostatic pressure-flow module, the systemic flow and mean arterial pressure were set to 5.0 L/min and 92 mmHg, respectively. Importantly, in this previous study,^13^ we demonstrated that this virtual HFpEF phenotype had reduced exercise tolerance, as characterized by a disproportional filling pressure response during exercise (mLAP/CO = 3.4 WU).

Second, considering the high prevalence of AF in the study cohort and HFpEF in general, an additional virtual HFpEF patient cohort was simulated with atrial contractile dysfunction by reducing active myofiber contractile function to 50%. This second virtual patient simulation allows to investigate the isolated effects of atrial myopathy on the response to accelerated atrial pacing rates.

Next, accelerated atrial pacing was simulated in the two reference virtual HFpEF patient simulations, in with and without of AV sequential pacing. Accelerated atrial pacing was simulated by gradual increases in pacing rate from 60 to 140 bpm in 10 bpm increments. Haemodynamic steady-state was ensured using the homeostatic pressure-flow regulation module to maintain constant systemic flow and mean arterial pressure regardless of pacing rate. To simulate AV sequential pacing, the PR interval, defined as the onset of atrial to ventricular activation in the model, was varied from 0 to 300 ms in 10 ms increments. Overall, this *in silico* trial allowed for a direct comparison of haemodynamic response to accelerated pacing over a wide variety of pacemaker settings in a virtual HFpEF cohort in the absence and presence of atrial myopathy.

### Statistical analysis

Statistical analysis was conducted using MATLAB 2019a with the Statistics and Machine Learning Toolbox 11.5. Continuous variables are presented as mean ± SD and SE or as median (interquartile range) according to distribution. Discrete variables are presented as count and proportion (%). Normality was assessed using the Shapiro-Wilk test. Paired differences were analysed using the paired Student *t-*test or the Wilcoxon signed-rank test, as appropriate, and are reported as mean ± SD with 95% confidence intervals. Two-sided *P*-values were reported, and a significance level of <.05 was considered significant.

## Results

### Study population

Of the 93 consecutive patients undergoing catheter ablation for AF enrolled in this study, 18 patients were excluded from the analysis. Eight of these patients were excluded due to persistent AF, while 10 patients were excluded because the pacing protocol was not completely followed. *Table 1* summarizes the clinical characteristics of the 75 patients in the study population, all of which free of AF events during the accelerated pacing procedure. The cohort consisted of 30 female (40%) elderly (66 ± 9 years) patients with a preserved LV ejection fraction of 57 ± 7% (with a 95% confidence interval ranging from [41–66]%), an increased LA volume indexed for body surface area of 37 ± 11 mL/m^2^, and 42(56%) patients with hypertension. Twenty-one patients (28%) were prescribed angiotensin-converting enzyme inhibitors or angiotensin receptor blockers, 52 (69%) were prescribed β-blockers, and 12 (16%) were prescribed calcium-channel blockers. Furthermore, NT-proBNP levels were 211 [90–525]pg/mL. Moreover, while no additional structural and function HFpEF inclusion criteria were defined, 49 (65%) patients had an H_2_FPEF score ≥5.0 points, suggesting a high HFpEF likelihood (>.80) in the study population, and 28 (37%) patients had an invasively measured elevated mLAP ≥15 mmHg in sinus rhythm.

**Table 1:** Characteristics of the study population

Patient data	Patient population (*n* = 75)
Age (years)	66 ± 9
Female, *n* (%)	30 (40%)
BMI (kg/m^2^)	28 ± 5
BSA (m^2^)	2.0 ± .2
Hypertension, *n* (%)	42 (56%)
NT-proBNP (pg/mL)	211 [90–525]
**Medications**	
ACEi/ARB, *n* (%)	21 (28%)
Beta-blockers, *n* (%)	52 (69%)
Calcium channel blockers, *n* (%)	12 (16%)
**LV and LA geometry**	
LV end-diastolic diameter (mm)	51 ± 6
LV end-systolic diameter (mm)	35 ± 5
Interventricular septum thickness (mm)	8.6 ± 1.3
Posterior wall thickness (mm)	8.6 ± 1.2
Relative wall thickness	.35 ± .06
LV mass (g)	159 ± 45
LV mass/BSA (g/m^2^)	78 ± 19
LA volume (mL)	76 ± 25
LA volume/BSA (mL/m^2^)	37 ± 11
**Functional indices**	
LV ejection fraction (%)	57 ± 7
E (cm/s)	72 ± 22
E/A—ratio	.9 [.0–1.2]
E/e′ septal	8.7 ± 3.0
Tricuspid regurgitant velocity (m/s)	2.2 ± .6
**HFpEF**	
H2FPEF class	5.2 ± 1.4
Invasive mLAP ≥ 15 mmHg at baseline, *n (%)*	28 (37%)

A, peak active atrial diastolic transmitral velocity; ACEi/ARB, angiotensin-converting enzyme inhibitor/angiotensin receptor blocker; BMI, body mass index; BSA, body surface area; E, peak passive diastolic transmitral velocity; e′, peak annular motion during E wave; LA, left atrial; LV, left ventricular; NT-proBNP, N-terminal pro-B-type natriuretic peptide.

### Acute haemodynamic and electrical response to accelerated atrial pacing

**Figure 2:**
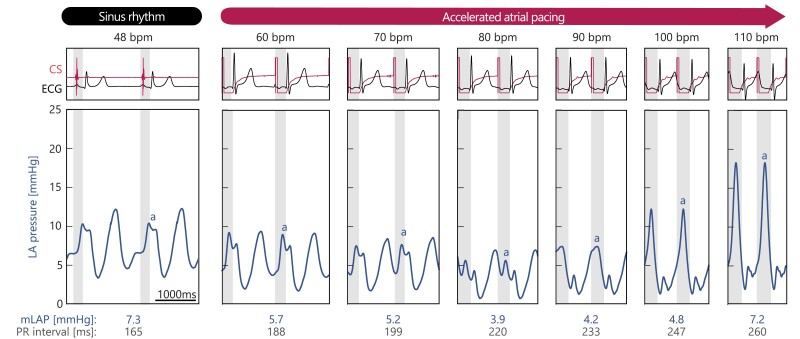
Patient example of the acute haemodynamic effects of accelerated atrial pacing. (Top row) Coronary sinus and electrocardiogram, and (second row) left atrial pressure recordings from a representative patient with the shaded areas indicating PR interval. A-wave annotation indicates left atrial systole, as guided by the pacing-spike detected on the coronary sinus catheter

*Figure 2* demonstrates the acute electrical and haemodynamic response to accelerated atrial pacing in a single patient. Initially, the patient presented with a heart rate 48 bpm in sinus rhythm and 7.3 mmHg mLAP. As the atrial pacing rate increased, mLAP gradually declined to a minimum of 3.9 mmHg at a pacing rate of 80bpm, herein referred as the observed patient-specific ‘optimal’ pacing rate. However, increasing the pacing rate further led to a subsequent rise in mLAP, ultimately surpassing the mLAP recorded in sinus rhythm when the pacing rate exceeded the 110 bpm. Concurrently, the PR interval consistently prolonged with increasing pacing rates. Notably, at higher pacing rates with severely prolonged PR intervals, ventricular repolarization, and relaxation were significantly delayed, leading to its merging or complete coincidence with atrial depolarization and subsequent contraction in the succeeding beat. This phenomenon elucidates the marked elevation of atrial pressure observed at high pacing rates (*Figure 2*).

**Figure 3:**
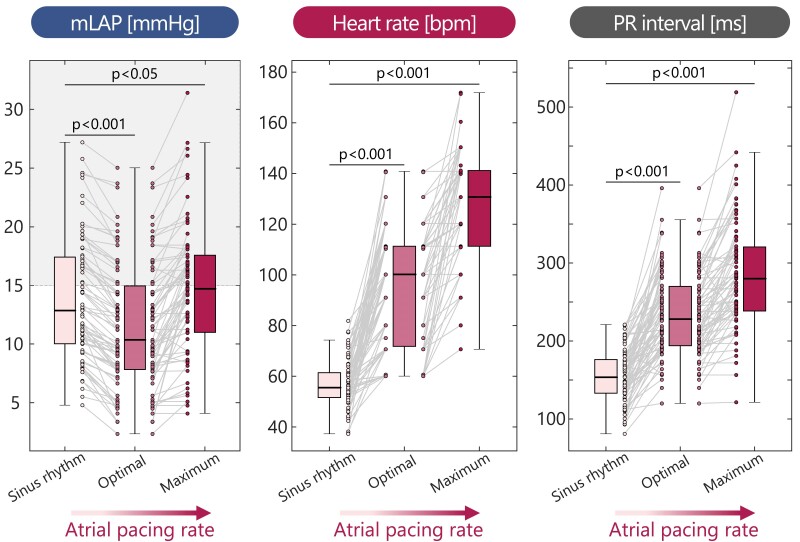
Clinically observed (*n* = 75) changes in mean left atrial pressure, heart rate, and PR interval as induced by accelerated atrial pacing. The ‘optimal’ category is the patient-specific pacing rate resulting in the lowest mean left atrial pressure observed during the accelerated atrial pacing. The ‘maximum’ category is the pacing rate prior to the rate which induced Wenckebach block. The shaded area indicates elevated mean left atrial pressure ≥15 mmHg

The haemodynamic and electrical effects of accelerated atrial pacing in the total patient population is shown in *Figure 3*. Starting from sinus rhythm, mLAP was 12.8 [10.0–17.4] mmHg with a heart rate of 55 [52–61] bpm. Accelerated atrial pacing significantly reduced mLAP by −2.3 ± 1.4 mmHg (*P* < .001), with a 95% confidence interval of −2.6 to −1.9 mmHg, to 10.4 [7.8–14.8] mmHg at the optimal pacing rates of 100 [70–110] bpm (*P* < .001). Notably, all patients demonstrated a decrease in mLAP, indicating efficacy irrespective of elevated mLAP ≥ 15 mmHg at sinus rhythm. Conversely, higher pacing rates of 130 [110–140] bpm (*P* < .001) resulted in an increase of mLAP to 14.7 [11.0–17.6] mmHg (*P* < .05), with 44 (59%) patients surpassing the recorded mLAP at sinus rhythm. Lastly, accelerated atrial pacing-induced decremental AV conduction, as evidenced by PR interval prolongation from 153 ± 20 ms at sinus rhythm to 228 ± 34 ms (*P* < .001) and 280 ± 45 ms (*P* < .001) at the patient-specific optimal and maximum pacing rates, respectively.

### Computer simulations

#### Effect of accelerated pacing on left-heart haemodynamics

**Figure 4:**
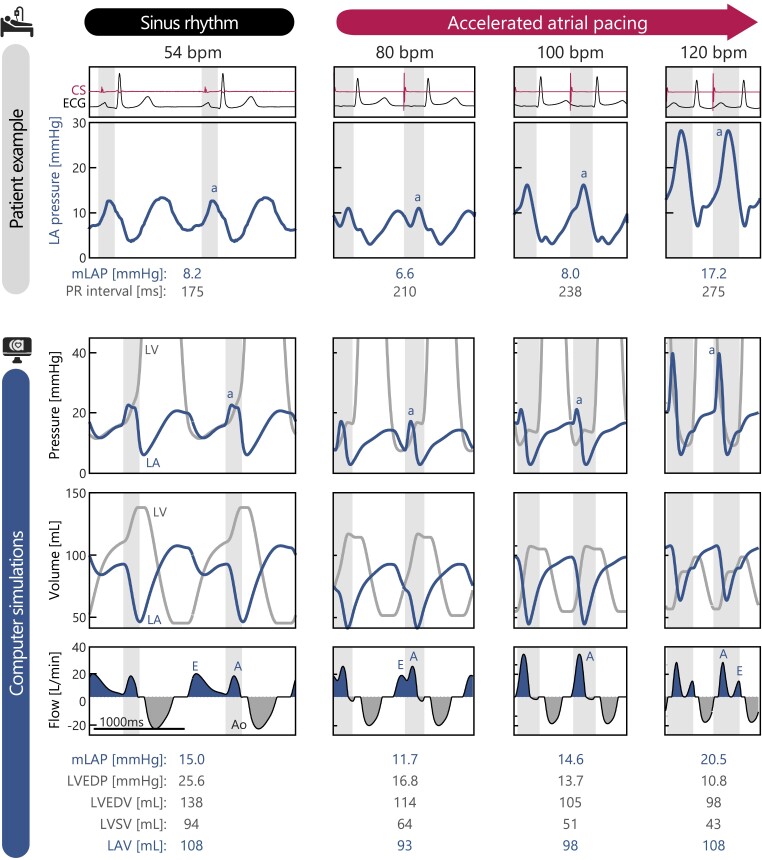
Comparison between a patient example and computer simulation of the acute haemodynamic effects during accelerated atrial pacing. (Top row) Coronary sinus catheter and electrocardiogram, and (second row) left atrial pressure recordings from a representative patient with the shaded areas indicating PR interval. (Third row) LV and left atrial pressures, (fourth row) volumes, and (last row) mitral and aortic flow as obtained from computer simulations. A-wave annotation indicates left atrial systole, as guided by the pacing-spike detected on the coronary sinus catheter (clinical data) or the moment of atrial mechanical activation (computer simulation). A, peak diastolic flow during atrial systole; Ao, aortic flow; E, peak diastolic flow by ventricular relaxation; LVEDP, left ventricular end-diastolic pressure; mLAP, mean left atrial pressure

*Figure 4* demonstrates the acute haemodynamic changes as observed in a single patient as compared with the computer simulations with a key phenotypic HFpEF features. The simulations, which had matching heart rate and PR interval, exhibited good qualitative agreement with the clinically measured LA pressure traces. This agreement was evident in both timing and morphology of the pressure traces throughout the cardiac cycle. Additionally, the simulations offered further insights into the haemodynamic effects of accelerated pacing which were not measured clinically, including simultaneous recording on LV diastolic pressure (third row), LA and LV volumes (fourth row), as well as mitral and aortic flow (bottom row). Here, it is observed that accelerated pacing continuously reduced LV end-diastolic pressure from 25.6 mmHg (intrinsic heart rate) to 10.8 mmHg at maximum pacing rate, along with LV end-diastolic volume from 138 to 98 mL and stoke volume from 94 to 43 mL. Interestingly, although the LV diastolic pressure exhibited a continuous reduction, simulations revealed that this reduction did not result in a corresponding decrease in LA pressure and volume at higher pacing rates. Analysis of the mitral flow pattern revealed that at higher pacing rates with prolonged PR intervals LA contraction coincides with early LV relaxation. This resulted in atrial contraction against a closed mitral valve resulting in a high-amplitude pressure wave that is transmitted backwards into the venous circulation, often referred to as a ‘cannon A-wave’. The simulations show that this phenomenon observed in the clinical pressure recording directly arises from the AV dissociation following the physiologic decremental AV conduction. This elucidates the mechanism underlying the marked increase in mLAP at higher pacing rates observed in the study population.

#### Effect of AV coupling on accelerated pacing response

**Figure 5:**
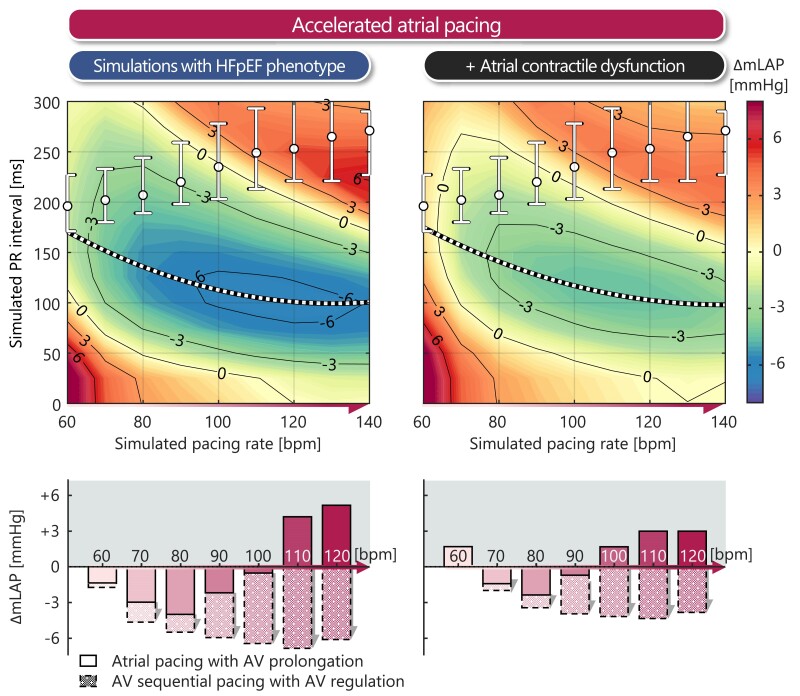
Change in mean left atrial pressure as function of pacing rate and PR interval in the virtual heart failure with preserved ejection fraction patient simulations with atrial contractile (dys)function. The upper panels show continuous effects of pacing rate and PR interval on changes in mean left atrial pressure compared with simulation with heart failure with preserved ejection fraction phenotype at intrinsic heart rate (i.e. mean left atrial pressure of 15 mmHg). The PR intervals as measured in the patient population (circles with whiskers, median [interquartile range]), as well as the model-predicted optimal PR interval (dashed line), at each pacing rate are projected on top of the simulation data. The lower panels show the quantitative mean left atrial pressure changes during accelerated pacing derived from the co-ordinates in the heatmaps corresponding to clinically observed PR interval relationships (represented by solid bars) and model-predicted optimum (depicted as dashed bars)

*Figure 5* shows the relative change in mLAP compared with the reference virtual HFpEF simulation, where mLAP is 15 mmHg intrinsically, in response to different pacing rates and PR intervals.

First, the continuous heatmaps demonstrate that higher pacing rates with long PR intervals (top-right) and lower pacing rates with short PR intervals (bottom-left) are associated with a detrimental increasing in mLAP up to +6 mmHg. The abovementioned simulation results have shown that this increase in mLAP follows from the atrium contracting against a closed mitral valve, either during early ventricular diastole (long PR intervals) or during ventricular contraction (short PR intervals). Moreover, by calculating the model-predicted optimal (i.e. lowest) mLAP for each pacing rate (dashed line), it can be observed that shortening the PR interval through AV sequential pacing results in a greater reduction in mLAP compared with clinically observed decremental AV conduction with accelerated atrial pacing rates [circles with whiskers, median (IQR)]. The bar graph demonstrates that without AV sequential pacing, the greatest mLAP reduction occurred at 80 bpm with a PR interval of 207 ms, resulting in reductions of −3.9 and −2.4 mmHg in the absence and presence of atrial contractile dysfunction, respectively. With AV sequential pacing, the lowest mLAP was observed at 110 bpm with a PR interval of 100 ms, leading to reductions of −6.1 and −4.4 mmHg in the absence and presence of atrial contractile dysfunction, respectively. This pronounced reduction in mLAP with AV sequential pacing is attributed to the improved AV coupling, which allows for more effective active atrial contribution to ventricular filling by avoiding atrial contraction against a closed mitral valve or high ventricular diastolic pressure (*Figure 4*).

Second, atrial contractile dysfunction attenuates mLAP unloading response to accelerated atrial pacing rates, regardless of pacing rate and PR interval. Moreover, the virtual HFpEF simulations with atrial dysfunction more closely resembled the mLAP pacing response as observed in the patient population with AF.

## Discussion

In this unique translational study, integrating clinical observations in patients with AF and an *in silico* trial evaluating the haemodynamic effects of various pacing strategies in a virtual HFpEF patient cohort, we demonstrated the potential of accelerated atrial pacing to reduce left-heart filling pressure. First, the clinical study revealed the acute haemodynamic and electrical response to accelerated atrial pacing in patients undergoing catheter ablation for AF, who were free of AF rhythm during the study procedure. Second, complimentary integrative computer simulations representing a virtual HFpEF patient cohort provided mechanistic insights potentially underlying the observed clinical outcomes. These simulations were instrumental in hypothesizing factors that modulate the efficacy of accelerated pacing, such as AV conduction delay and atrial contractile (dys) function.

The main result of this study is that the optimal pacing rates of 100 [70–110] bpm acutely reduce mLAP (−2.3 ± 1.4 mmHg), but induces decremental AV conduction (PR interval prolongation) which, subsequently, leads to mLAP increase at higher pacing rates. The virtual HFpEF patient cohorts suggest that by improving AV coupling through AV sequential pacing (PR interval regulation) the beneficial effects of accelerated atrial pacing can be further optimized. Furthermore, the absolute reduction in mLAP was more substantial in the computer simulations as compared with the clinical study, likely due to the more pronounced HFpEF phenotype present in the simulations than in the average patient in the clinical cohort.

While alleviating left-heart filling pressure is a central therapeutic aim in HFpEF management,^17^ currently therapeutic paradigms predominantly consider heart rate lowering rather than acceleration. This is especially true for patients with AF, in whom heart rate reduction is strongly recommended, even between AF episodes. Our findings have important translational implications for future *in vivo* investigations into the relative merits of heart rate and/or AV coupling modulation for improving outcomes in patients with HFpEF.

### Therapeutic implications

Our study has several important therapeutic implications as it provides insights into the mechanism by which accelerated atrial pacing rates reduce left-heart filling pressure. First and foremost, both the findings in patients (*Figure 3*) and the virtual HFpEF patient cohorts (*Figure 5*) demonstrate that increasing atrial pacing rate can acutely reduce mLAP. Reducing filling pressure is important not only to reduce congestion-related symptoms but also for decreasing atrial wall stress, a factor that has been associated with AF.^18^ Indeed, the computer simulations demonstrate that reducing mLAP was associated with reduced maximum LA volume and, therefore, atrial wall stress (*Figure 4*). However, increasing atrial pacing rates beyond the patient-specific optimal pacing rate resulted in a decremental increase in mLAP, which even exceeded the value at intrinsic heart rate during sinus rhythm. Hence, our study challenges the effectiveness of a one-rate-fits-all approach, particularly given the high interpatient variability in optimal pacing rate (*Figure 3*) and advocate for a more personalized accelerated pacing protocol considering patient-specific heart rate in sinus rhythm, AV coupling, and underlying cardiovascular (dys)function.

Second, the patient population showed a significant acute reduction in mLAP of −2.3 ± 1.4 mmHg at the 100 [70–110]bpm optimal pacing rate (*Figure 3*). This reduction is similar to the reported change in PCWP of −2.5 ± 3.7 mmHg observed in the CAMEO-DAPA trial,^19^ which evaluated the efficacy of sodium-glucose cotransporter-2 inhibitors. In addition to PCWP reduction, this trial also demonstrated significant improvements in hospitalizations and mortality. Furthermore, our observed mLAP reduction also aligns with the findings of the REDUCE LAP-HF trial,^20^ which used an interatrial shunt device to alleviate elevated LA pressure. While our preliminary results are promising, future clinical research is needed to further establish clinical relevance. Importantly, our clinical observations demonstrated a reduction in mLAP that was independent of the left-heart filling pressure status in sinus rhythm, indicating its potential therapeutic relevance across the spectrum of HFpEF severity.

Third, pharmacological heart rate lowering with beta-blockers or ivabradine is standard practice in managing heart failure patients, however, their efficacy in HFpEF has been debated.^21–23^ Our study results corroborate the paradigm-changing hypothesis that moderate heart rate acceleration, rather than heart rate lowering, might alleviate symptoms in patients with HFpEF.^24^

### Accelerated pacing induces negative AV dromotropy

In accordance with previous studies,^5,25^ we observed a significant PR interval lengthening with increasing atrial pacing rate (*Figures 2* and *3*). This observation likely stems from multiple factors. Firstly, proximal CS pacing induces non-physiological atrial activation, characterized by LA preexcitation and reversal of the activation wavefront along the annulus.^26^ This reverse activation pattern utilizes fewer intervascular conduction fibres compared with sinus activation, potentially prolonging intra- and interatrial activation times. This effect may be exacerbated by local structural or electrophysiological abnormalities typically observed in atrial tissue of AF patients, further slowing or blocking the activation wavefront.^27^ Secondly, atrial pacing fundamentally differs from physiological (exercise-induced) heart rate increase, as it lacks the accompanying adrenergic surge that normally enhances conduction velocity and shortens refractory periods.^28^ This absence of adrenergic modulation may be further compounded by anaesthesia, beta-blockers, and/or calcium channel blockers used by most patients in our cohort (*Table 1*), all of which are known to suppress adrenergic responses.

These factors could collectively attenuate mLAP reduction through prolonged total atrial activation time and impaired AV coupling. The altered atrial activation pattern and lack of adrenergic modulation would likely result in delayed arrival of the depolarization wave at the AV node. This delay, combined with potential changes in atrial and AV nodal properties, contributes to the observed PR interval prolongation with increasing pacing rates, potentially limiting the haemodynamic benefits of accelerated pacing.

### Haemodynamic improvements related to AV sequential pacing

A crucial finding of this study is that the beneficial haemodynamic effects of accelerated pacing can be further optimized by AV sequential pacing, by preventing PR interval prolongation at higher atrial pacing rates and thereby preserving AV coupling (*Figure 5*). Although this observation was not tested in our clinical cohort, several studies have demonstrated the haemodynamic advantages of modulating AV coupling.^29–31^ Furthermore, it is a known physiological response that the PR interval decreases with increasing heart rate during exercise.^32^ Moreover, it can be hypothesized that the dependence on appropriate AV coupling becomes increasingly significant with exercise intensity, where both heart rate and cardiac output increase, thereby reducing ventricular filling time and necessitating greater reliance on active atrial contribution to ventricular filling.

Recently, Salden *et al*.^15^ observed that in patients with prolonged PR intervals (>200 ms), as well as in animals and computer simulations of AV dromotrophy, PR interval shortening at rest led to an acute increase in LV stroke volume and cardiac output, along with lower LA pressures. These haemodynamic benefits were attributed to improved LV filling function, due to larger and better separated passive (E) and active atrial (A) mitral inflow, as compared with shortening of filling time and fusion of the E-wave and A-wave at prolonged PR intervals. Our computational simulations presented in the current study support these findings on the haemodynamic benefit of properly timed atrial contraction and extend its importance across various pacing rates.

While previous studies have demonstrated the benefit of accelerated atrial pacing,^4^ the feasibility, safety, and long-term efficacy of heart rate acceleration with AV sequential pacing has not yet been studied and therefore requires further investigation through prospective clinical trials. The exact strategy by which AV sequential pacing is achieved, however, warrants consideration, as traditional RV apical pacing has been reported to adversely affect cardiac function and effective diastolic duration due to abnormal ventricular electrical activation.^33,34^ Hence, alternative pacing locations, such as more physiological biventricular or conduction system pacing^35^ could be explored to minimize potential pacing-induced dyssynchronopathy and facilitate sufficient diastolic time at higher pacing rates.^36^

### Atrial (dys) function modulates left-heart unloading response

Alongside evaluating the influence of AV coupling, our study investigated the modulating effect of intrinsic atrial contractile function on reducing left-heart filling pressure by accelerated atrial pacing, particularly considering the presence of AF in our study cohort and its prevalence in the HFpEF population in general.^37^ While this aspect could not be explored clinically, computer simulations allowed for a direct comparison of pacing outcomes with and without atrial contractile dysfunction, demonstrating an mLAP reduction regardless of atrial dysfunction (*Figure 5*). Indeed, in both our study as well as the myPACE clinical trial^4^ the prevalence of AF in the patient population was significant, while both observed positive outcomes of increased pacing rates. Specifically, 31% of patients in the myPACE trial had pacemaker-detected AF at baseline, which at 1-year follow-up reduced to 18%. Our results provide potential mechanistic evidence underlying this improvement by demonstrating that the lower left-heart filling pressure with accelerated atrial pacing leads to reduced atrial and ventricular diastolic pressures as well as reduced peak atrial stretch, all of which known are substrates for AF alleviation.^18,37^ Moreover, our study hypothesizes that the presence of atrial myopathy does not contraindicate accelerated pacing as a therapeutic strategy to reduce left-heart filling pressure, but rather that it does so with less efficacy as compared with normal atrial function.

While the simulation assumed atrial contractile dysfunction to arise from intrinsic mechanical atrial myopathy, such as AF, abnormal electrical atrial activation has also been associated with reduced atrial contractile function.^27,38^ Indeed, prolonged intra-atrial delay is highly prevalent in HFpEF patients, indicated by increased P wave duration, particularly with dilated atria,^39^ which would suggest a shorter left-heart PR interval. Recent studies have shown that different atrial pacing locations affect total atrial activation times and, consequently, atrial contractile function.^40,41^ In addition, Bachmann's Bundle area pacing has been demonstrated to lead to the most synchronous atrial activation compared with right atrial appendage and CS pacing.^42^ Given our observation that atrial (dys)function modulates the absolute reduction in left-heart filling pressure with accelerated pacing, further investigation is required to determine the potential influence of different atrial pacing locations.

### Future perspectives

Since the study's patient selection was limited to those undergoing AF ablation, it does not fully represent the broader HFpEF population, despite the notable likelihood of HFpEF in the cohort and elevated filling pressure in sinus rhythm (*Table 1*). Given the high heterogeneity of underlying cardiovascular disease in HFpEF, it is reasonable to anticipate that accelerated atrial pacing with or without AV sequential pacing may not uniformly benefit all patients. Silvermann *et al*.^43^ explored whether increasing atrial pacing acutely induces detrimental Ca^2+^-handling, potentially compromising ventricular relaxation and filling function. They observed increased myocardial Ca^2+^-retention in HFpEF patients, suggesting enhanced ventricular diastolic function at moderately increased rate. Nevertheless, future clinical studies are needed to investigate the feasibility and benefit of AV sequential pacing at accelerated rate in a more representative HFpEF patient population.

It is important to interpret the computational results with caution when applying them to clinical situations. First, we modelled a virtual HFpEF cohort characterized by reduced active ventricular relaxation and increased passive stiffness, with and without atrial myopathy. These virtual cohorts exhibited more pronounced HFpEF features than the study cohort and captured key structural and functional HFpEF features—such as preserved LV ejection fraction, diastolic dysfunction, and reduced exercise tolerance.^13^ Future computational studies should investigate a wider range of HFpEF subgroups to enhance the generalizability of our findings. Second, the simulations assumed homeostatic pressure-flow regulation to maintain constant systemic flow and mean arterial pressure regardless of pacing rate, PR interval, or cardiovascular function. However, in our clinical study patients various cardiovascular, pharmacological, or anaesthesia-induced homeostatic regulatory modulations could be expected.^44^ Consequently, it is reasonable to infer that unlike the computer simulations, physiological homeostatic pressure-flow regulation may not occur during the accelerated atrial pacing protocol in all patients. Therefore, future clinical studies should consider monitoring systemic arterial pressure or cardiac output in addition to LA pressure. This will enable differentiation between: (i) reduction of left-heart filling pressure due to a decrease in cardiac output, (ii) reduction in filling pressures with minimal or no change in cardiac output, and (iii) minimal change in filling pressure but significant increase in cardiac output—where only the latter two responses could be considered beneficial, be it by reducing backwards failure or enhancing forward function.

## Conclusion

By integrating clinical observations and computer modelling this study demonstrated the mechanism by which accelerated atrial pacing reduces left-heart filling pressure and revealed important factors by which therapy response is modulated. The clinical study elucidates the acute haemodynamic response to accelerated atrial pacing in patients with AF, characterized by a significant left-heart filling pressure decrease at the optimal pacing rates as compared with sinus rhythm, followed by an unfavourable increase of filling pressure and prolongation of AV conduction delay at higher pacing rates. The simulated virtual patient cohorts offered comprehensive insights into underlying dynamics and modulating factors of the pacing therapy, hypothesizing on the potential of AV sequential pacing to further optimize the left-heart unloading response by preserving AV coupling. Overall, our study challenges current therapeutic paradigms and underscores the importance of exploring heart rate acceleration and AV coupling modulation as potential therapeutic intervention in patients with HFpEF and/or AF.

## Supplementary data

Supplementary data are not available at *European Heart Journal* online.

## Declarations

### Data Availability

The data underlying this article will be shared on reasonable request to the corresponding authors.
